# Natural serine proteases and their applications in combating amyloid formation

**DOI:** 10.5599/admet.2551

**Published:** 2024-11-16

**Authors:** Sanjay Kisan Metkar, Saranya Udayakumar, Agnishwar Girigoswami, Koyeli Girigoswami

**Affiliations:** 1 Medical Bionanotechnology, Faculty of Allied Health Sciences, Chettinad Hospital and Research Institute, Chettinad Academy of Research and Education, Chettinad Health City, Kelambakkam, Chennai-603103, India; 2 Centre for Global Health Research, Saveetha Medical College, Saveetha Institute of Medical and Technical Sciences, India

**Keywords:** Amyloidosis, lumbrokinase, serratiopeptidase, nattokinase, prion disease, Alzheimer’s disease

## Abstract

**Background and purpose:**

Amyloidosis is a group of diseases including diabetes type II and neurological disorders, such as Alzheimer’s disease, Parkinson’s disease, prion disease, etc., where a common trait is observed; accumulation of misfolded protein at different parts of the body, especially the brain which manifests the typical symptoms like dementia, movement disorders, etc. These misfolded proteins, named amyloids, are protease resistant and thus it becomes difficult to manage these diseases in vivo. Enzymes that catalyse the complete breakdown of proteins are known as proteases. The peptide bonds in proteins are degraded by these serine proteases, which cause amyloid disaggregation.

**Experimental approach:**

We have searched for related articles using the search engines Google Scholar, PubMed, and Scopus for the past 10 years, selected the relevant articles, and written the outcomes and benefits of protease using the medical topic “serine protease” and the following text phrases -keratinase, lumbrokinase, serratiopeptidase, nattokinase.

**Key results:**

Alkaline serine proteases exhibit activity within the neutral to alkaline pH range. They are most capable of degrading host complement proteins, cytokines, and host clotting factors mostly due to their serine centre or metallotype. Because of its potential usage in food, pharmaceutical, and other industrial domains, this category of enzymes has been extensively investigated. Specifically, serine proteases are a group of enzymes that can be consumed orally and are stable in our gastrointestinal tract.

**Conclusion:**

In this review, we discussed the role of different serine proteases in amyloid aggregate inhibition and their potential application in treating amyloidosis.

## Introduction

Amyloidogenic proteins are well known for their ability to misfold and aggregate normal proteins into insoluble fibrils. It plays a crucial role in the pathogenesis of several neurodegenerative diseases, including Alzheimer's, Parkinson's, Huntington's, and other diseases like diabetes type II. This aggregation of amyloid fibrils disrupts cell function, resulting in gradual tissue impairment. To degrade these aggregates, the body employs a range of proteolytic enzymes. Serine proteases have created widespread interest due to their broad substrate specificity and potent catalytic activity [1]. On the other hand, evidence exists that amyloids can be degraded *in vitro* using zinc oxide nanoflowers [2] and nanoformulated carrageenan [3].

Hydrolytic enzymes, such as proteases, phytases, lipases, amylases, amidases, and others, dominate the enzyme market today. Most of the proteases belong to the serine protease family and are characterized by a serine residue [4]. Serine protease acts as an endopeptidase by breaking down peptide bonds like other proteins. However, these residues in active sites play a crucial role in coordinating functions through protein hydrolysis [5]. Moreover, these types of protease are widely distributed in every living organism, including viruses, bacteria, fungi, insects, and humans, and function both within and outside the cells [6]. Serine proteases are traditionally classified into various categories, including chymotrypsin-like, trypsin-like, subtilisin-like, elastase-like, and thrombin-like protease. Chymotrypsin-like proteases can be found in both prokaryotes and eukaryotes. Over 240 chymotrypsin-like proteases have been identified in the MEROPS peptide database. Chymas, synthesized by mast cells, play essential roles in various peptide hormones and other physiological processes, such as hemostasis, inflammation, apoptosis, signal transduction, immunological responses, and digestion. It also breaks down the peptide bonds on the carboxyl side of tryptophan, phenylalanine, and tyrosine. This particular type of protease sequentially activates blood clotting, fibrinolysis and wound healing [7]. Trypsin-like protease is a homologous protease found in the human genome. Out of 699 proteases in humans, 138 belong to the S1 protease family and the other 178 are serine proteases [8]. This trypsin-like protease performs a key function in boosting the body’s response to blood clots, protein digestion, and immunity [9]. Subtilisin is a serine protease found in prokaryotes. According to the MEROPS database, subtilases are a subfamily of the clan SB of serine peptidases and belong to S8A family. Generally, they act as a detergent to remove proteinaceous dyes. Compared to other protease types like trypsin and chymotrypsin, elastase-like protease has a smaller S1 cleft. These proteases cleave bonds at the non-polar amino acids such as alanine, glycine, and valine [5,10]. Snake venoms contain numerous highly toxic thrombin-like serine protease isoforms. It consists of two chains linked by disulfide bonds: the A and B chains. It is involved in fibrinolysis, and Alzheimer's and Parkinson's-induced dementia and other neurodegenerative conditions [5,11].

**Figure 1.**
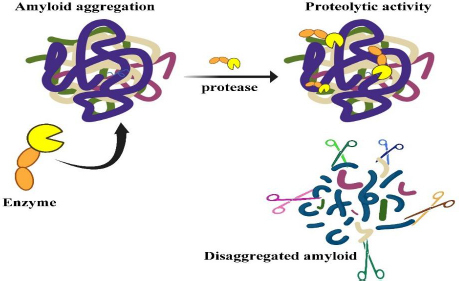
Action of protease in amyloid degradation

Among those serine proteases, lumbrokinase (LK) and serratiopeptidase (SP) are the world's most exciting enzymes being studied for their potential application in the medical field [5]. A new dimension has emerged with the discovery of nattokinase (NK) and keratinase (KER), which are unique proteases that are capable of destroying recalcitrant proteins like amyloid, known as protease-resistant proteins [12,13]. The mechanism of protease in the clearance of amyloid misfolded proteins is depicted in Figure 1.

## Specific types of serine proteases

### Lumbrokinase

Since ancient times (<4000 years, 2600 B.C.), earthworms have been closely linked with human beings. They have been utilized as food and medicine to treat various human ailments in different parts of the world, including China, Japan, Korea, India, Cambodia, Myanmar (Burma), Vietnam, Iran, and the Middle East [14]. The Greek philosopher Aristotle (384-322 B.C.) mentioned the importance of earthworms and termed them “The Intestine of Earth” [15-17]. They also have been used for sore throat, healing wounds, piles, chronic boils, and so forth for an exterior application, and traditional treatment of internal diseases like chronic cough, diphtheria, jaundice, rheumatic pains, tuberculosis, bronchitis, facial paralysis, and impotence [18].

The epithelial cells in the gizzard segment, especially in the anterior alimentary regions, express and produce earthworm fibrinolytic proteases. In these parts, the proteases possibly play a role in digesting protein and peptides in food [19]. LK was isolated chiefly from *Lumbricus rubellus*, and *Eisenia fetida*. Earthworm protease enzymes (EPE) and fibrinolytic enzymes (EFE) are some names given to LK [20]. In some cases, proteases are named after the Latin binomial for the species of earthworm from which they are derived. For example, *E. fetida* protease (Efp) is a protease extracted from *E. fetida*. Several fibrinolytic enzymes were extracted from *L. rubellus*, including F-1-0, F-1-1, F-I-2, F-II, F-III-1, and F-III-2 [21]. Zhou *et al.* purified seven fibrinolytic proteins from *E. fetida* in 1988 [22]. LK isozymes were also extracted from *L. biomass* and *E. Andrei*. LK can sustain its activity in both basic and acidic conditions, having a wide pH range from 1 to 11. The molecular weight of LK lies between 20 and 35 kDa, with a range of isoelectric pH (pI) from 3 to 5 [21]. Moreover, some LK are resistant to high temperatures and can withstand up to 60° C. The same fibrinolytic enzyme has multiple names because each enzyme was isolated and named independently by different research groups, making accurate determination of the total number of LK difficult. Nomenclature for LKs should be standardized according to their function, properties, and sources [23]. Conventional approaches to LK extraction and earthworm purification are complex and time-consuming. In order to assess the potential clinical application of LK protein, researchers used recombinant DNA technology to express and characterize a single LK protein [24]. The plasminogen activation system mechanism of LK differs from other thrombolytic enzymes, such as streptokinase, urokinase, staphylokinase, recombinant tissue-type plasminogen activator, and recombinant prourokinase. LK is not only involved in the activation of plasminogen but can also activate fibrin directly [25]. These enzymes execute predominantly proteolysis of fibrinogen and fibrin and hardly hydrolyse other plasma proteins, such as plasminogen and albumin [25,26].

According to the data available in GenBank, eight clones of accessible cDNA of LK (GenBank Accession No.; AY187629, U25644, AY438622, AY178854, AF304199, U25648, AF433650) are available. The coding sequence of LKs cDNA has a length of 852 bp and encodes 283 amino acids, in which 36 amino acid codes for the signal peptide and the remaining 247 amino acids code for the whole protein [24,27,28]. The nucleotide arrangement in each clone of cDNA starts with 13 codons containing "CG" motifs in the complete sequence, which is almost rare for mammals. This makes the translation of such DNA in mammalian cells or tissues inefficient [29]. A cDNA of gene EFE-3 of *Eisenia fetida* has 859 independent nucleotides, with an open reading frame accessible from 112-853, which encodes a polypeptide with 247 amino acid residues [27].

The sequence of LK isolated from different earthworm species was analysed to get the full proteomics data. The protein sequence from *L. rubellus* and *E. fetida* had more residues than the secondary structures, including α-helix, turn, β-sheet, and coil. The arrangement of an LK isoform known as earthworm fibrinolytic protease II (Ef P-II (EFEa)) is remarkably identical with other serine proteases with its known structures [30, 31] or serine protease related to earthworm [29]. The earthworm protease catalytic characterization is directly affected by its tertiary structure. The studies on NMR and X-ray analysis revealed that Ef P-III-1 (EFE-b) belongs to the group of trypsin-like protease possessing two chains- a light chain at the N-terminal of pyroglutamate and an N-glycosylated heavy chain [32]. The structural aspect showed Ef P-III-1 having high stability regarding heat resistance and resistance to proteases and organic solvents [33]. Further, it was noted that another LK isoform, Ef P-II is a serine protease type chymotrypsin possessing an essential S1 binding pocket [31].

**Figure 2.**
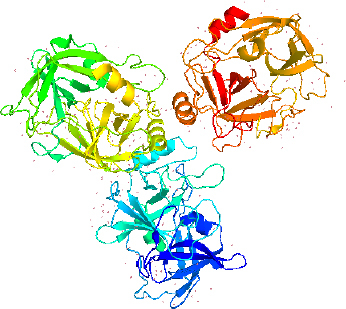
The structure of Lumbrokinase (Source: PDB id 1M9U) extracted from PyMol.

LK structure extracted from PyMol with PDB id 1M9U, as depicted in Figure 2, showed the chymotrypsin-like serine proteases with characteristics of S1 elastase. The S1 specificity of the beta strand significantly showed four different residues (Ser-Ser-Gly-Leu) after Val217, which can provide additional substrate for the hydrogen binding sites for distal P residues and gives elongation for the S1 pocket. The S1 pocket prefers elastase-specific small hydrophobic, strong, and bulky P1 residues that support the tight substrate binding and induced S1 pocket fitting. This structure was first labelled to represent earthworm fibrinolytic enzyme components as well as serine protease, which originated from the annelid worms [34].

### Fibrinolytic and proteolytic activities of lumbrokinase

Lumbrokinase (LK) has been utilized as a fibrinolytic agent in China, Japan, and Korea. The arrangement of thrombus inside the vessels of blood causes numerous inconveniences, for example, myocardial infarction and stroke. A considerable lot of local and recombinant proteins with proteolytic properties have been utilized as effective thrombolytic agents, such as urokinase (UK), streptokinase, recombinant tissue-type plasminogen activator, recombinant prourokinase, and staphylokinase [35-38]. A considerable lot of them have indicated great outcomes. However, they additionally have a few confinements, for example, quick clearance, the absence of protection from reocclusion, bleeding complications, and other adverse impacts [39]. LK has both functions, like plasminogen activation and fibrinolysis and has been used to treat thrombosis [40]. When consumed orally, the animal experiments showed that LK had a significant fibrinolytic efficacy. Additionally, distinct improvement was witnessed in the management of high blood viscosity syndrome and thrombocytosis [41]. Moreover, LK is relatively stable under long-term storage at ambient temperature. As an oral medication, LK is known to avert and treat clotting diseases, including cerebral thrombosis and myocardial infarction [42]. Specifically, *Lumbricus rubellus* and *Lumbricus bimastus*, are the essential resources for a fibrinolytic agent in Southeast-Asian nations, for example, China, Korea, Japan and so on [43, 44]. The isolation and purification of EFEs included centrifugation, filtration, ammonium sulphate precipitation and dialysis, ion exchange chromatography, and affinity chromatography [45], which made the scaling up difficult. A simple method was employed to scale up, like aqueous-two phase systems (ATPS), which included separation and the target protein concentration, giving a clear extract [46]. Seven types of purified fibrinolytic enzymes (EFE-a to EFE-g) and eight glycosylated purified fibrinolytic enzymes (EfP-0-1 to EfP-III-2) were identified from the source of *Eisenia fetida* till today [21,47,48].

Six fractions of lumbrokinase (F-I-0 to F-III-2) having fibrinolytic activity are purified from *Lumbricus rubellus* lysates using autolysis, fractionation with ammonium sulphate and column chromatography. Lumbrokinase’s F-III-1 and F-III-2, F-I-1and F-I-1 both showed a similar activity although they have different molecular weights [23]. Table 1 summarizes the various physiological and biological properties of these enzymes.

**Table 1.** The biological and physiological properties of different EFEs [43]

Organism	Enzyme name	Enzyme activity	Physiological properties	Biological properties
*Eisenia fetida*	EfP-0-1 to EfP-III-2	Relative activity: 6.2, 12.8, 25.8, 31.6, 8.8, 2.1, 12.5, 2.3 % (the activity of mixture as 100 %). EfP-0-2 and EfP-II-2 were newly isolated	Trypsin-like fibrinolytic enzymes in glycosylated form	EfP-II and EfP-III-1 were strongly inhibited by SBTI
	Lumbrokinase	Fibrinolytic and thrombolytic activity	No data	Proteins with isoelectric point in the range 4.6 to 7.4
	EFE-a to EFE-f	EFE-b, EFE-c, and EFE-g had relatively higher fibrinolytic activity EFE-d and EFE-e had average fibrinolytic activity, and EFE-a and EFE-f had relatively lower activity EFE-a has plasminogen-activating activity and fibrinolytic activity	EFE-b, EFE-c, and EFE-g represent trypsin-like enzymes, EFE-d, EFE-e and EFE-f represent chymotrypsin-like enzymes. EFE-a unknown	Acid enzymes
*Eisenia fetida*	PI and P II (tissue homogenate (G-90) of *Eisenia foetida*)	All with the esterase activity, but only fraction PI displayed amidase activity, and the majority of activity was represented by PI	They belong into the tyrosine family	PMSF at the concentration of 10 to 4 M inhibited PI (BAEE as substrate)
	ARSPI	A plasmin and also a plasminogen activator	Glycoprotein or glycopeptide	Inhibited by PMSF
*Lumbricus rubellus*	Isozymes A to F (formerly named F-III-2, F-III-1, F-II, F-12, F-I-1 and F-I-0, respectively)	They have acted on elastin, fibrin, and actual fibrin clots of whole blood in a rat’s vena cava. They also catalysed the hydrolysis of various esters. Especially A and B have much higher strong caseinolytic and fibrinolytic activities than plasmin. The enzymes have also strong amydolytic activity.	Trypsin-like serine proteases with single polypeptide chains and are not glycoproteins. Isozymes A, B, D, E, and F represent both trypsin- and chymotrypsin-like activities, but isozymes C also serves as an elastase-like enzymes	The enzymes were stable at below 60 °C, over a range of pH 2 to 11, most active at 55 °C, on heating at 80 °C for 30 min, the activity completely disappeared they retain full activity for long years at room temperature. High stability toward organic solvents and detergents, the activity of isozymes A, B, and C was inhibited strongly by soybean trypsin inhibitor and aprotinin, but the enzyme activity of D, E, and F was partially inhibited by these inhibitors.
	F1 to F6	The rank activity orders of proteolytic activities and fibrinolytic activities are F2> F1> F5>F6> F3>F4> and F6> F2>F5>F3>F1>F4 separately	All of them are serine protease, F5 A and F6 are trypsin-like serine protease. Iso-enzymes. F1 is chymotrypsin-like protease.	F1-F4 were completely inhibited by PMSF. F5 and F6 were completely inhibited by aprotinin, TLCK, TPCK, SBTI LBTI, and leupeptin. The six iso-enzymes were stable at pH 4 to-12.

There has been an increase in the use of the LK protein in the field of medicine today, mainly due to its ability to perform thrombolytic therapy. Because LK is a eukaryotic protein, it is even more important in the treatment of cardiovascular and cerebrovascular diseases. LK has been produced both in bacterial and eukaryotic systems as a result of advances in recombinant technology. As a result of advanced design of the appropriate host system and alteration of the genetic level of a drug to make it more effective, there have been attempts to improve the expression of the recombinant enzyme, immobilization for reuse of the enzyme, and chemical modification in order to reduce antigenicity and improve its activity in order to make a drug more effective. It is expected that it will be the right drug candidate in the future for the treatment of thrombosis [49]. Moreover, an *in vivo* study demonstrated that the fibrinolytic enzyme from earthworm has reduced intra-abdominal adhesion and peritoneal thickening [50].

### Role of lumbrokinase in cerebral Ischemia and Implantation

By increasing the level of c-AMP and reducing calcium release from calcium reserves, LK has anti-ischemic activity due to its antiplatelet activity. LK had an antithrombotic effect due to the inhibition of expression of ICAM-1 and an antiapoptotic effect due to the activation of the JAK1 / STAT1 pathway, which causes the antithrombotic effect. As a result of the antithrombotic and antiapoptotic functions of the Janus Kinase 1 / signal transducers and transcriptional activators 1 (JAK1 / STAT1), the brain was protected from ischemic damage through the action of ICAM-1 and Janus Kinase 1 on the brain's intercellular adhesion mechanisms [51]. When an artificial organ is transplanted, a small thrombus usually forms on the surface of the graft, which may lead to serious complications, such as the rejection of the graft if the thrombus is not removed. Despite the fact that there has been considerable achievement in the field of medicine, there is still a concern about the compatibility of the organ with the improvement in blood circulation, which leads to offensive results in transplantations. Maleic anhydride-methyl vinyl ether (MA-MVE) has been used as a catalyst for immobilizing the LK on polyurethane and, as a result, has shown high anti-thrombogenic activity, and has reduced the formation of surface-induced thrombi. There is a possibility that an immobilized LK surface may reduce the adhesion and activation of platelets by inhibiting fibrinogen adsorption or by altering the conformation of adsorbed fibrinogen at an early stage of blood contact by decreasing their adhesion and activation [52]. The oral administration of LK led to a significant increase in the levels of silent information regulators (Sirt1), respectively. The study report suggested that the post-ischemic treatment with LK from earthworm mitigated myocardial IR injury by stimulating Sirt1 and, enhancing autophagic flux, and reducing IR-induced injury [53]. Another study conducted by Wang *et al.* evaluated that LK (10 μg/kg) exhibits protective effects against heart disorder in a rat model [54]. This collection of evidence could successfully conclude that LK has promising applications in biomedicine.

## Serratiopeptidase

Serratiopeptidase (SP) (EC number 3.4.24.40) is an active proteolytic enzyme found in enterobacteria *Serratia marcescens* (Gram-negative bacteria) and belongs to the family of alkaline serine protease. It has a molecular weight of 45 kDa to 60 kDa and comprises three zinc atoms as ligands at the active site [55]. SP cannot bind proteins in healthy tissues since its chemical structure prevents it from doing so [56]. SP depends on secretory protein on the membrane for its production, and the N-terminal signal peptide-independent pathway secretes it [57]. SP is considered by high-level specificity towards its substrate. The fibrinolytic enzyme activity of SP is due to its activity in the degradation of insoluble proteins, such as fibrin and other mediators of inflammation [58]. They were also used to treat several chronic disorders like arthritis, atherosclerosis, bronchitis, fibrocystic breast disease, carpal tunnel syndrome, Crohn’s disease, traumatic swelling, leg ulcers, fibromyalgia, migraine, breast engorgement, sinusitis, lung disorders, hepatitis, diabetes, thrombosis, carotid artery blockage, and uterine fibroids [59,60]. This enzyme is available as a supplement to enhance the cardiovascular system and overall health. The enzyme SP is also considered to be a healing enzyme because it heals sprained muscles, traumatic injuries, leg ulcers, and torn ligaments, drains mucus, postoperative inflammation, and reduces the viscosity and elasticity of nasal mucus [61].

**Figure 3.**
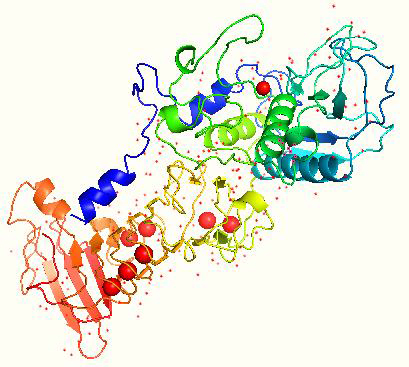
The crystal structure of SP generated by using PyMol [PDB ID-1SAT].

The gene encoding SP is composed of 470 amino acids. SP amino acid sequence is free from sulfur, methionine, and cysteine [62] with a G + C content of 58 % [63]. The maximum SP activity is at pH 9.0 and a temperature of up to 40 °C [58]. The saturation point where SP is thoroughly degraded was found to be at 55 °C [62]. The isoelectric point of SP is found to be 5.3 [64]. The gene sequence of SP is cloned and sequenced, and its crystal structure has been determined, as shown in Figure 3 [63].

*Serratia marcescens* HR-3, SP gene was expressed in *E. coli* (DE3) / pLysS using the pET32a (+) expression vector. It has been reported that the enzyme is highly expressed as inclusion bodies and that purified SP contains no activity [65]. Similarly, the SP gene from *Serratia marcescens* strain SM6 was allowed to express in *E. coli* by using the *lac* promoter, which secreted the enzyme into the medium, but the enzyme was found to be inactive with a high molecular weight [66]. The structural gene of SP is not clustered with the secretion genes, which makes improper processing of the zymogen protease that is dysfunctional in the *E.coli* expression system, making the recombinant SP inactive [67]. Further, the SP gene was cloned into the pPICZαA Pichia expression vector and electrotransformed into the *Pichia pastoris* GS115, outputting the maximum expression at 72 h using the *E.coli* expression system. Yeast can be a suitable alternative host for such types of secretory proteins [31].

### Fibrinolytic activity

SP is known to be safe as it is regularly taken by the oral route as a fibrinolytic drug and has the potential to circulate in the blood due to its ability to be absorbed by the intestine [68,69]. The therapeutic use of SP may lead to less bioavailability because of its lower membrane permeability and high tendency to undergo degradation by the enzymes present in the gastrointestinal tract [69]. In rats, after taking a dose of 100 mg/kg of SP, SP can be detected in the serum and lymph at concentrations of 0.87 and 43 ng/mL, respectively [32,33]. The exact bioavailability data of SP in humans is not yet reported anywhere in the literature [69].

Fibrin can repair injuries caused by trauma, surgery and damage to old cells, replacement by new cells, tissues, and muscles [70]. The blood vessels of the damaged tissue form a composite substance called thromboplastin, which binds to the broken blood vessels of the tissue and emits a platelet factor if the blood vessels are damaged. A Prothrombin activator is created when thromboplastin and platelet factor react with calcium ions and other elements to form an insoluble fibrin that blocks blood flow and oxygen supply in the body, causing infarction of myocardium, stroke, pulmonary embolism, and vein clots [59]. Thus, fibrinolytic enzymes like SP, which dissolve clots, are crucial to the human body to avoid the considerable risk of damage. Mei *et al.* [71] demonstrated strong thrombolytic effects in *in vitro* model, with 96.6 % clot lysis at a concentration of 300 U/mg of SP. Therefore, the study indicates that SP could be a suitable candidate for both the prevention and therapy of thrombotic disorders [71].

### Anti-inflammatory effect of serratiopeptidase

Most diseased conditions produce a certain level of inflammation, which causes hostility in the disease. This inflammation aggravates the immune system to activate white blood cells (WBCs), which travel via the circulatory system to destroy pathogenic bacteria and foreign substances and kills cancer cells. During the repair mechanism, the WBCs can translocate into organs or tissues, and such elevated activity of WBCs results in cell damage [61]. SP was initially utilized for its anti-inflammatory properties in Japan in 1957 [56]. Khateeb *et al.* [72] examined the anti-inflammatory impact of SP on orthodontal inflammatory syndrome. In general, pathogen-induced inflammation can manifest as either acute or chronic inflammation. Acute inflammation is a protective response to infection, while uncontrolled resolution leads to chronic inflammation [72]. The remarkable anti-inflammatory effects of SP and diclofenac in acute and subacute inflammation were evaluated by Jadav *et al.* [73]. Nonsteroidal anti-inflammatory drugs (NSAIDs) are recommended for acute inflammation, while NSAIDs, in combination with steroidal drugs and SP, are used to address chronic inflammation [74]. Recent investigations established that SP combined with paracetamol can successfully reduce edema and acute inflammation in rat models. Hence, this study also suggests that SP could be a promising candidate for anti-inflammatory drugs [75]. A study by Krishna *et al.* also highlighted the anti-inflammatory activity of SP, demonstrating its effectiveness in reducing postoperative swelling compared to dexamethasone [76]. The role of SP in vascular inflammation induced by lipopolysaccharide (LPS) was also investigated. The result showed that SP reduced LPS-induced oxidative stress in mice and decreased the expression and activity of monocyte chemoattractant protein-1 (MCP-1) [77]. The author evaluated the effectiveness of two drug combinations for reducing postoperative pain and swelling after the surgical removal of impacted mandibular third molars. In this study, two drug combinations were orally administered to sixty patients three times continuously for three days. The drug combination of trypsin, rutoside, and bromelain showed less swelling. Whereas diclofenac and serratiopeptidase drug exhibited higher swelling and less pain. Hence, the study concluded that bromelain drug is effective in reducing swelling, and diclofenac, SP-based therapy provides superior pain relief [78]. The anti-inflammatory effect of SP ointment was evaluated in mice. Compared to control group, SP ointment treated mice demonstrated significant anti- inflammatory effect [79].

### Clinical applications of serratiopeptidase

SP reduces pain by arresting the discharge of pain, which influences amines such as bradykinin from inflamed tissues [80]. SP has the capacity to cause hydrolysis of histamine, serotonin, and bradykinin, which are accountable for eliciting oedemic responses [81]. The substrate bradykinin binds in the vicinity of the zinc-binding site of SP, which efficiently cleaves the peptide bond of bradykinin [82]. At the infection site, they modulate the transformation of cell adhesion molecules, which are intricate in the inflammatory regulatory cells. SP is taken orally to control the inflammation that occurs due to sinusitis, prostate gland inflammation, carpal tunnel syndrome, breast engorgement, chronic emphysema, and acute and chronic ear-nose-throat disease [55]. In 2021, Gupta and colleagues illustrated the efficacy of using SP and vitamin D together to combat the severe consequences of COVID-19 conditions [83]. During the pandemic, COVID-19 has led to various respiratory diseases, including nasal congestion and cough among patients. Various studies reported that mucolytic drugs can help boost bronchial mucus secretion or reduce mucus viscosity [55]. Furthermore, a comparison of SP’s mucolytic activity with seaprose indicated that proteolytic enzyme were active in *in vivo* animal models [84]. Gioia’s 2020 study on patients with respiratory disease found that SP enhanced mucus clearance by lowering the neutrophils, which changed sputum viscosity. This shows that SP could be an effective therapy for respiratory problems and COVID-19 consequences [85].

The bacterium thrives by making biofilm within the microbiome. These biofilms usually form surfaces of the bacteria; for example, *Staphylococcus aureus*, which has a variety of virulence factors and possesses the potential to invade the eukaryotic cells by making surface biofilm and dominates with *staphylococcal* infections. Blocking of such *Staphylococcus aureus* colonization can inhibit the quick spread of biofilm [86].

The anti-infective effect of SP is exerted through inert surface attachment, thereby inhibiting Staphylococcus adherence and ceasing invasion of eukaryotic cells. The detailed mechanism is yet to be explored. SP has also shown an antibacterial effect against *Escherichia coli* and *Pseudomonas aeruginosa* [87,88]. Mecikoglu *et al.* [89] conducted an *in vivo* study that used SP to treat bacterial infections. 94.4% of the infected rats treated with SP recovered, showing its potential in combating biofilm-forming activity. Using proteolytic enzymes like SP in combination with antibiotics could be effective against microbes [89].

## Keratinase

**Figure 4.**
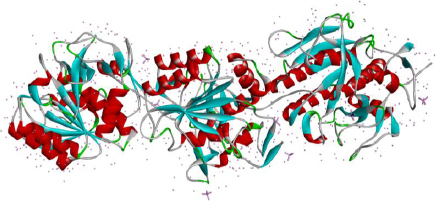
The structure of Keratinase (Source: PDB id 5wsl) extracted from PyMol [98].

Keratinases (KER) are extracellular enzymes usually produced from *Bacillus, Streptomyces, Aspergillus, Fervido bacterium, Xanthomonas, Chryseo bacterium*, and *Vibrio*, which are known to degrade keratin protein in eukaryotes. KER is especially known for its use in de-hairing leather before processing and in the cosmetic industry [90,91]. Prion disease or transmissible spongiform encephalopathy (TSE) is a neurological disease manifested due to the aggregation of misfolded proteins called prions. Prion protein gets transmitted from infected animals through the consumption of cattle feed prepared from dried brains and carcasses of dead infected animals [92,93]. The types of TSEs in humans are CJD, transmissible mink encephalopathy (TMC) in mink and kuru [94]. A reported novel application of KER (also known as prionzyme) is the hydrolysis of the pathogenic form of prion protein, which is known to be resistant to any other protease [95]. The present prion treatment methods comprise its inactivation using chemicals and heat, which has limitations regarding environmental acceptability, application and cost compatibility, making way for enzyme inactivation of prion [96]. KER-mediated prion protein destruction is feasible at a nominal cost and is eco-friendly [97]. The structure of keratinase is extracted from the Protein database and given in Figure 4.

### Amyloidogenic activity of keratinase

Amyloid β peptides (Aβ) are poisonous peptides composed of 39 to 43 amino acid residues. When Aβ aggregates are deposited in brain tissues, they block synaptic transmission and cause neurodegenerative illnesses. Several experiments have been done to disintegrate these hazardous aggregates. Amycolatopsis Ker1 and Ker2 were tested for their ability to break down Aβ fibrils. In this study, Aβ fibrils of lysozymes were treated with varying doses (50, 100, 125, 250, 500 mg/ml) of keratinase, and their degraded structure was analysed over time intervals. At 24 h, Ker1 had a larger effect than Ker2 [99]. In recent years, numerous studies have suggested that keratinolytic enzymes could potentially be utilized in degrading Aβ aggregates and prion protein (PrP^Sc^). Keratinase MSK103 from *Bacillus licheniformis* efficiently degraded PrP^Sc^ in brain tissue homogenates infected with scrapie in 2 h at 50 °C. After 20 hours of decontamination, prions were reduced to lower levels. Hence, it proves that MSK103 keratinase can also degrade PrP^Sc^ [100]. The researchers discovered that the combination of *B. licheniformis* N22 keratinase and *Pseudomonas aeruginosa* NCIMB 8626 biosurfactant formulation was capable of partially degrading ME7 scrapie prions after treating them for 1 h at 50 °C. When the temperature increased from 50 to 65 °C, the PrP^Sc^ proteins became undetectable in just 10 min. Interestingly, it was noted that the *B. licheniformis* N22 keratinase alone could not completely break down the ME7 scrapie prions even after extending the period for 1 h. However, the biosurfactant NCIMB 8626 combined with enzyme N22 keratinase showed complete degradation of infectious prion protein [101]. KER isolated from the moderate thermophile *B. licheniformis* PWD-1 strain was the first described enzyme that was capable of disintegrating pretreated prion (*i.e*., heated at 115 °C for 40 min along with SDS) [102]. The purified version of KER (strain MSK 103) showed the degradation and decontamination of prion-infected brain homogenates at 50 °C without prior heat treatments with detergents. This KER has shown intense activity in the 60-70 °C range with pH 9 to 10 [103]. Using an alkaline serine protease isolated from Streptomyces 99-GP-2D-5 strain, scrapie prion degradation could be achieved within 3 min. At a temperature of 60 °C and a pH level of 11, the enzyme has been shown to exhibit its maximum activity [104]. Therefore, most studies concluded that combination therapy of keratinase enzyme has enhanced anti-amyloidogenic activity against Aβ and prion [105]. The limitation of keratinase action is the high temperature it needs to degrade the amyloids in most cases, which cannot be achieved under physiological conditions. Keratinase can be used for surface sterilizations and sterilizations of surgical equipment.

## Nattokinase

**Figure 5.**
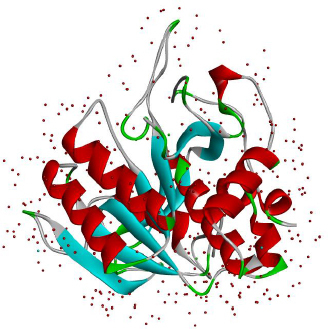
The structure of Nattokinase (Source: PDB id 4dww) extracted from PyMol [114].

The consumption of boiled soybeans has been practiced for almost 100 years in different Asian regions. The microbe present in fermented natto is a Gram-positive bacteria that can also produce endospores and is named *Bacillus subtilis* natto (formerly known as *Bacillus* natto) [106,107]. Nattokinase (NK) is known as Subtilisin NAT, an extracellular enzyme produced by *Bacillus subtilis* natto [108]. NK comes into the serine protease family with the catalytic triad that is conserved with three residues like Asp-32, His-64, and Ser-221 [109]; it has an isoelectric point at pH 8.7, and the molecular weight is about 27.7 kDa [110]. NK is made up of 275 amino acids, and its gene sequence is identical with various members of the subtilisin family (86 % with subtilisin BPN, and 72 % with Carlsberg subtilisin type, 99.5 % homology with subtilisin E). It disintegrates fibrin in thrombi as well as cleaves plasminogen activator inhibitor type I [111]. The structure of nattokinase from the protein data bank is shown in Figure 5.

NK supersedes plasmin toward thrombolytic activity [112], and oral administration causes absorption from the intestinal tract to induce fibrinolysis. This makes NK a potential anticoagulant for cardiovascular disease treatment and coronary artery disease [113]. The nutritional supplement natto helps to destroy the intima of arteries thickening and aids in the breakdown of mural thrombi seen as a result of endothelial injury [66].

The results showed a reduction in fluorescence emission at 487 nm of Thioflavin-T (ThT) dye (a dye that specifically binds to amyloids), negative ellipticity at 218 nm in Circular dichroism (CD) spectrum, and less fibril morphology in electron microscopic observation. Overall, this study explored the potential of NK for the degradation of amyloid at a temperature of 40 °C and pH 7 [12].

### Fibrinolytic of nattokinase

Nattokinase (NK) has been recognized for its fibrinolytic activity by disintegrating amyloid at normal body temperature and neutral pH. This makes it a promising candidate for treating amyloid-related diseases and other neurodegenerative diseases. Numerous studies have reported that both *in vitro* and *in vivo* NK has neuroprotective properties [115]. For instance, oral administration of NK has been shown to be effective against cerebral ischemia, resulting in reduced infarct quantity in gerbils due to enhanced fibrinolytic activity [116]. The enzyme NK not only dissolves blood clots but also breaks down Aβ. The study result showed fibrinolytic activity in NK that has lowered factor VII and factor VIII [117]. The study conducted by Guangbo *et al.* revealed that the NK extracted from *B. subtilis* has significant fibrinolytic activity. The recombinant NK displayed a clear thrombolytic effect and exhibited strong pH and thermostability [118]. NK has exhibited strong clot-dissolving capabilities and a low risk of bleeding. It reduces clot lysis time and lowers the indicators linked to cardiovascular disease [119]. The NK extracted from *B. subtilis* G8 has shown excellent fibrinolytic activity [120].

Kamiya *et al.* found that the NK enzyme reduced the length of carrageenan-induced rat tail and the mass of ferric chloride-induced carotid thrombus [121]. The cationic peptide from natto extract exhibited excellent angiogenic activity in human umbilical vein endothelial cells, indicating the potential of NK as an antithrombotic agent [122]. Another study reported that sustained release of synthesized NK-conjugated magnetite nanoparticles was identified as a potential candidate for thrombolysis [123]. A recent study showed the application of NK towards amyloid degradation by efficiently degrading the three types of *in vitro* prepared amyloid fibrils-insulin, Aβ1-40, and huPrP (consisting of prion sequence 108-144). The amyloids were generated using these peptides in appropriate conditions and were taken to explore the degradation capacity of NK. The results showed a reduction in fluorescence emission at 487 nm of Thioflavin-T (ThT) dye (a dye that specifically binds to amyloids), negative ellipticity at 218 nm in Circular dichroism (CD) spectrum, and less fibril morphology in electron microscopic observation. Overall, this study explored the potential of NK for the degradation of amyloid at a temperature of 40 °C and pH 7 [12]. NK not only plays a role in fibrin degradation but also enhances the release of tissue plasminogen activators from cells to break down the fibrin [124].

### Anti-inflammatory effect

In recent studies, an anti-inflammatory agent, like NK, is effective in suppressing various inflammatory diseases and oxidative stress (ROS). Wu *et al.* demonstrated that NK can suppress LPS-induced NOX2 activation and the TLR4 pathway to reduce ROS and proinflammatory mediators [125]. Moreover, long-term use of NK has shown promising results in ameliorating chronic colitis and inhibiting colonic inflammation by regulating gut microbiota and suppressing the TrP metabolism [126]. Additionally, NK exhibits an inhibitory effect against retinal neovascularization and can alleviate neuroinflammation by inducing proinflammatory microglia into an anti-inflammatory phenotype through the Nrf2/HO-1 pathway [127]. Another study by Elbakry *et al.* suggested that NK can protect against neurotoxicity and attenuate neuroinflammatory activity induced by certain substances. Notably, most clinical studies have indicated that short-term and low-dosage administration of NK did not induce toxicity [128].

### Nattokinase’s role in other disease

Research has identified natural anticancer compounds such as phytoestroprotease inhibitors, flavonoids, and phytic acids in soybeans (natto). Furthermore, studies have shown that fermented natto contains higher levels of anticancer compounds compared to the raw material. According to Chou *et al.* [129], both natto freeze drying and natto water extract have been found to trigger cell autophagy in a dose-dependent manner. Hypertension stands as the most prevalent chronic illness and a significant risk factor for cardiovascular disease. NK from *Ruditapes philippinarum* decreased blood pressure in hypertensive rats by minimizing intestinal bacterial diseases, providing an effective defense against hypertension-induced vascular and cardiac damage [130]. Furthermore, studies indicated that oral administration of NK has decreased systolic and diastolic blood pressure by cleaving fibrinogen in plasma. Moreover, as per Keziah, NK exhibited notable angiotensin-converting enzyme inhibitor activity, achieving 87.45 % in an *in vitro* coagulation lysis assay. These findings suggest that NK plays a significant role in preventing and treating hypertension [131]. Similar findings were also demonstrated by another enzyme SP [132].

## Lumbrokinase and serratiopeptidase in amyloid degradation

Prion protein PrP^C^ is a cellular protein post-translationally modified into a transmissible, prion-associated protein known as PrP^Sc^. As a result of conformational changes in the PrP 106-126 region of the PrP^Sc^ peptide, fibrillation is initiated in this protein and thus, PrP 106-126 can be used for studying prion peptide amyloidosis. There is a possibility that any agent capable of destabilizing or disintegrating such proteins can be used as a potential drug candidate for the treatment of prion diseases. The activity of LK and SP was *in vitro* tested against PrP 106-126 amyloids, which was measured against NK, the standard amyloid degrading enzyme. Based on ThT fluorescence assay results, both LK and SP inhibited PrP 106-126 amyloid formation. Furthermore, after incubation of prion amyloids with LK and SP, fibril sizes were also found to be smaller at different time intervals using dynamic light scattering. Furthermore, the molecular dynamics simulation revealed that PrP 106-126 had a high affinity for LK and SP. According to the study, SP and LK have the potential to disintegrate PrP 106-126 amyloids and improve the viability of the PC12 cells [133]. A similar result was also found for the amyloid β 1-42, the protein that gets misfolded and deposited in the brain in case of Alzheimer’s disease [134]. In vitro studies of the degradation of insulin amyloid using LK and SP and the results were promising [132, 135]. When the study was extrapolated in an animal model, where we induced insulin amyloid mass, after repeated subcutaneous injection of insulin amyloids, we could observe that the insulin amyloid formation was retarded and the amyloids were degraded using both LK and SP [136].

## Other natural compounds reported as amyloid inhibitors

Natural compounds with anti-amyloid potential are always more beneficial than synthetic ones as they are routinely consumed. Various polyphenols like epigallocatechin-3-gallate (EGCG), resveratrol and curcumin are reported for anti-amyloid potential and progressed for clinical trials. To date, 72 natural compounds are reported for the inhibition of amyloids and 44 compounds belong to a phenolic group, 4 anthraquinones, 16 flavonoids, 13 alakoloids (includes 3 pyridines, 2 porphyrins, and 3 indoles), terpenes, and steroids. Cyclodextrin, squalamine, vitamin A, hematin, rifampicin, and scyllo-inositol have anti-amyloid activity shown under *in vitro* conditions [137,138]. There are several agents reported as amyloid inhibitors, some of them are in clinical trials, as shown in Table 2 [139].

**Table 2.** Anti-amyloid drugs in the pipeline for clinical trials.

Stages	Agent	Agent mechanism class	Mechanism of action	Therapeutic purpose	ClinicalTrials.gov ID [139]	Ref.
Phase I	Aducanumab	Anti-amyloid	Monoclonal antibody	Remove amyloid (DMT)	NCT01677572	[140]
	Crenezumab	Anti-amyloid	Monoclonal antibody	Remove amyloid (DMT)	NCT02353598	[141]
	KHK6640	Anti-amyloid	Anti- Aβ peptide antibody	Remove amyloid (DMT)	NCT03093519	[142]
	LuAF20513	Anti-amyloid	Polyclonal antibody	Remove amyloid (DMT)	NCT02388152	[143]
	LY3002813	Anti-amyloid	Monoclonal antibody	Remove amyloid (DMT)	NCT02624778	[144]
Phase II	BAN2401	Anti-amyloid	Monoclonal antibody	Remove amyloid (DMT)	NCT017673311	[145]
	E2609	Anti-amyloid	BACE inhibitor	Reduce amyloid production (DMT)	NCT02322021	[146]
	Octagam 10 %	Anti-amyloid	10 % human normal immunoglobulin	Remove amyloid (DMT)	NCT03319810	[147]
	UB-311	Anti-amyloid	Active immunotherapy	Reduce amyloid (DMT)	NCT0255180	[148]
Phase III	CNP520	Anti-amyloid	BACE inhibitor	Reduce amyloid production (DMT)	NCT03131453	[149]
	Gantenerumab	Anti-amyloid	Monoclonal antibody	Remove amyloid (DMT)	NCT02051608	[150]
	MK-8931 (verubecestat)	Anti-amyloid	BACE inhibitor	Reduce amyloid production (DMT)	NCT01953601	[151]
	Solanezumab	Anti-amyloid	Monoclonal antibody	Remove amyloid (DMT)	NCT02008357	[152]

Further research is warranted to determine many more drugs that can cross the blood-brain barrier and effectively degrade the amyloids responsible for various neurodegenerative disorders.

## Conclusion

Biotechnology has become highly versatile and has merged with other technologies to deliver high-throughput products. Its amalgamation with bionanotechnology has created tremendous demand in the biomedical field. Enzymes from natural sources have been consumed in different forms worldwide for a long time, owing to their health benefits. Among the various serine proteases, SP, LK, KER, and NK have shown their potential in inhibiting the amyloid fibril formation responsible for different neurodegenerative disorders, such as Alzheimer’s disease, Prion disease, and type II diabetes. Early diagnosis of amyloidosis permits for effectively improved organ function. Moreover, if these natural inhibitors successfully complete clinical trials, outcomes for those suffering from amyloidosis can be improved, and serine protease enzymes may be used to improve quality of life in the early stages of the disease. In this mini review, we have discussed these serine proteases and other natural compounds that can be used to manage neurodegenerative diseases. These studies concluded that KER can be used to remove prion infections from surgical instruments and other surfaces contaminated with prions because KER is not consumable. On the other hand, LK and SP can be used to degrade amyloids. Since they possess fibrinolytic activity, they can be used as coatings above surgical implants so that they can retard the clot formation at the site of implant post-surgery. Future studies are necessary to nano-formulate these enzymes to effectively cross the blood-brain barrier and get delivered to the brain for exerting their anti-amyloid activity.

## References

[1] Metkar S.K. Udayakumar S. Girigoswami A. Girigoswami K.. Amyloidosis-history and development, emphasis on insulin and prion amyloids. Brain Disorders 13 (2024) 100106.https://doi.org/https://doi.org/10.1016/j.dscb.2023.100106. 10.1016/j.dscb.2023.100106

[2] Girigoswami A. Ramalakshmi M. Akhtar N. Metkar S.K. Girigoswami K.. ZnO Nanoflower petals mediated amyloid degradation-An in vitro electrokinetic potential approach. Materials Science and Engineering C 101 (2019) 169-178.https://doi.org/10.1016/j.msec.2019.03.086. 10.1016/j.msec.2019.03.086 31029310

[3] Udayakumar S. Metkar S.K. Girigoswami A. Deepika B. Janani G. Kanakaraj L. Girigoswami K.. Exploring the amyloid degradation potential of nanoformulated carrageenan-bridging in vitro and in vivo perspectives. International Journal of Biological Macromolecules 279 (2024) 134814.https://doi.org/https://doi.org/10.1016/j.ijbiomac.2024.134814. 10.1016/j.ijbiomac.2024.134814 39168227

[4] Jablaoui A. Kriaa A. Akermi N. Mkaouar H. Gargouri A. Maguin E. Rhimi M.. Biotechnological applications of serine proteases: a patent review. Recent Patents on Biotechnology 12 (2018) 280-287.https://doi.org/10.2174/1872208312666180924112007. 10.2174/1872208312666180924112007 30246645

[5] Patel S.. A critical review on serine protease: Key immune manipulator and pathology mediator. Allergologia et Immunopathologia 45 (2017) 579-591.https://doi.org/10.1016/j.aller.2016.10.011. 10.1016/j.aller.2016.10.011 28236540 PMC7126602

[6] Di Cera E.. Serine proteases. IUBMB life 61 (2009) 510-515.https://doi.org/10.1002/iub.186. 10.1002/iub.186 19180666 PMC2675663

[7] Hedstrom L.. Serine protease mechanism and specificity. Chemical Reviews 102 (2002) 4501-4524.https://doi.org/10.1021/cr000033x. 10.1021/cr000033x 12475199

[8] Page M.J. Di Cera E.. Serine peptidases: classification, structure and function. Cellular and Molecular Life Sciences 65 (2008) 1220-1236.https://doi.org/10.1007/s00018-008-7565-9. 10.1007/s00018-008-7565-9 18259688 PMC11131664

[9] Tyndall J.D. Nall T. Fairlie D.P.. Proteases universally recognize beta strands in their active sites. Chemical Reviews 105 (2005) 973-1000.https://doi.org/10.1021/cr040669e. 10.1021/cr040669e 15755082

[10] Barzkar N. Khan Z. Jahromi S.T. Pourmozaffar S. Gozari M. Nahavandi R.. A critical review on marine serine protease and its inhibitors: A new wave of drugs? International Journal of Biological Macromolecules 170 (2021) 674-687.https://doi.org/10.1016/j.ijbiomac.2020.12.134. 10.1016/j.ijbiomac.2020.12.134 33387547

[11] Stojanovski B.M. Pelc L.A. Di Cera E.. Thrombin has dual trypsin-like and chymotrypsin-like specificity. Journal of Thrombosis and Haemostasis 22 (2024) 1009-1015.https://doi.org/10.1016/j.jtha.2023.12.026. 10.1016/j.jtha.2023.12.026 38160728 PMC10960677

[12] Hsu R.-L. Lee K.-T. Wang J.-H. Lee L.Y.-L. Chen R.P.-Y. Amyloid-degrading ability of nattokinase from Bacillus subtilis natto. Journal of Agricultural and Food Chemistry 57 (2009) 503-508.https://doi.org/10.1021/jf803072r. 10.1021/jf803072r 19117402

[13] Mukherjee S. Ningthoujam D.S. Jaya Devi L. Laboratory M.B.R.. P4-033: Degradation of amyloid-beta aggregates by microbial keratinases. Alzheimer's & Dementia 11 (2015) P778-P778.https://doi.org/10.1016/j.jalz.2015.06.1737. 10.1016/j.jalz.2015.06.1737

[14] Yang W. Wang W. Ma Y. Yang Q. Li P. Du S.. Bioevaluation of Pheretima vulgaris Antithrombotic Extract, PvQ, and Isolation, Identification of Six Novel PvQ-Derived Fibrinolytic Proteases. Molecules 26 (2021) 4946.https://doi.org/10.3390/molecules26164946. 10.3390/molecules26164946 34443534 PMC8402109

[15] Sinha R. Herat S. Chauhan K. Valani D.. Earthworms: the'unheralded soldiers of mankind'and'farmer's friend'working day and night under the soil: reviving the dreams of Sir Charles Darwin for promoting sustainable agriculture. American-Eurasian Journal of Agricultural and Environmental Science 5 (2009) 5-13.https://doi.org/hdl.handle.net/10072/30153.

[16] Shipitalo M.J. Le Bayon R.-C. Quantifying the Effects of Earthworms on Soil Aggregation and Porosity, in Earthworm Ecology, Edwards C.A. (Ed.), CRC Press, Boca Raton, USA, 2004, 183-200.https://doi.org/10.1201/9781420039719.pt5. 10.1201/9781420039719.pt5

[17] Edwards C.A. Arancon N.Q. Bohlen P.J. Hendrix P., Biology and Ecology of Earthworms, Springer New York, NY, USA, 2022, ISBN 038774942X.https://doi.org/10.1007/978-0-387-74943-3. 10.1007/978-0-387-74943-3

[18] Afreen S. Shaikh A.. Therapeutic uses of earthworm–a review. International Journal of Advanced Ayurveda, Yoga, Unani, Siddha Homeopathy 9 (2020) 571-580.https://doi.org/10.23953/cloud.ijaayush.469. 10.23953/cloud.ijaayush.469

[19] Sumi H. Nakajima N. Mihara H.. A very stable and potent fibrinolytic enzyme found in earthworm Lumbricus rubellus autolysate. Comparative Biochemistry and Physiology Part B: Comparative Biochemistry 106 (1993) 763-766.https://doi.org/10.1016/0305-0491(93)90160-7. 10.1016/0305-0491(93)90160-7

[20] Stephani L. Rahayu P. Retnoningrum D. Suhartono M.T. Rachmawati H. Tjandrawinata R.R.. Purification and proteomic analysis of potent fibrinolytic enzymes extracted from Lumbricus rubellus. Proteome Science 21 (2023) 8.https://doi.org/10.1186/s12953-023-00206-9. 10.1186/s12953-023-00206-9 37158880 PMC10165752

[21] Wang F. Wang C. Li M. Gui L. Zhang J. Chang W.. Purification, characterization and crystallization of a group of earthworm fibrinolytic enzymes from Eisenia fetida. Biotechnology Letters 25 (2003) 1105-1109.https://doi.org/10.1023/a:1024196232252. 10.1023/a:1024196232252 12889822

[22] Nguyen Q.T.T. Rhee H. Kim M. Lee M.Y. Lee E.-J. Lumbrokinase, a Fibrinolytic Enzyme, Prevents Intra-Abdominal Adhesion by Inhibiting the Migrative and Adhesive Activities of Fibroblast via Attenuation of the AP-1/ICAM-1 Signaling Pathway. BioMed Research International 2023 (2023).https://doi.org/10.1155/2023/4050730. 10.1155/2023/4050730 PMC9851794 36685669

[23] Nakajima N. Mihara H. Sumi H.. Characterization of potent fibrinolytic enzymes in earthworm, Lumbricus rubellus. Bioscience, Biotechnology, and Biochemistry 57 (1993) 1726-1730.https://doi.org/10.1271/bbb.57.1726. 10.1271/bbb.57.1726 7764268

[24] Ge T. Sun Z.-J. Fu S.-H. Liang G.-D.. Cloning of thrombolytic enzyme (lumbrokinase) from earthworm and its expression in the yeast Pichia pastoris. Protein Expression and Purification 42 (2005) 20-28.https://doi.org/10.1016/j.pep.2005.04.005. 10.1016/j.pep.2005.04.005 15927482

[25] Verma M.K. Pulicherla K.. Lumbrokinase-a potent and stable fibrin-specific plasminogen activator. International Journal of Bio-Science and Bio-Technology 3(2) (2011) 57-69. https://gvpress.com/journals/IJBSBT/vol3_no2/5.pdf

[26] Munawar S. Sagir M. Mustafa G. Ali M.A. Niazi A.K. Parvaiz A. Yasmin F. Mansoor F. Kanwal S. Rasheed M.. In silico analyses of predicted substitutions in fibrinolytic protein ‘Lumbrokinase-6’suggest enhanced activity. Process Biochemistry 110 (2021) 292-301.https://doi.org/10.1016/j.procbio.2021.08.022. 10.1016/j.procbio.2021.08.022

[27] Dong G.-Q. Yuan X.-L. Shan Y.-J. Zhao Z.-H. Chen J.-P. Cong Y.-W.. Molecular cloning and characterization of cDNA encoding fibrinolytic enzyme-3 from earthworm Eisenia foetida. Acta Biochimica et Biophysica Sinica 36 (2004) 303-308.https://doi.org/10.1093/abbs/36.4.303. 10.1093/abbs/36.4.303 15253157

[28] Yuan X. Cao C. Shan Y. Zhao Z. Chen J. Cong Y.. Expression and characterization of earthworm Eisenia foetida Lumbrokinase-3 in Pichia pastoris. Preparative Biochemistry & Biotechnology 36 (2006) 273-279.https://doi.org/10.1080/10826060600716703. 10.1080/10826060600716703 16707338

[29] Sugimoto M. Nakajima N.. Molecular cloning, sequencing, and expression of cDNA encoding serine protease with fibrinolytic activity from earthworm. Bioscience, Biotechnology, and Biochemistry 65 (2001) 1575-1580.https://doi.org/doi.org/10.1271/bbb.65.1575. 10.1271/bbb.65.1575 11515541

[30] Takahashi L.H. Radhakrishnan R. Rosenfield Jr R.E. Meyer Jr E.F. Trainor D.A.. Crystal structure of the covalent complex formed by a peptidyl. alpha.,. alpha.-difluoro-. beta.-keto amide with porcine pancreatic elastase at 1.78. ANG. resolution. Journal of the American Chemical Society 111 (1989) 3368-3374.https://doi.org/10.1021/ja00191a039. 10.1021/ja00191a039

[31] Marquart M. Walter J. Deisenhofer J. Bode W. Huber R.. The geometry of the reactive site and of the peptide groups in trypsin, trypsinogen and its complexes with inhibitors. Acta Crystallographica Section B: Structural Science 39 (1983) 480-490.https://doi.org/10.1107/S010876818300275X. 10.1107/S010876818300275X

[32] Wang F. Wang C. Li M. Zhang J.-P. Gui L.-L. An X.-M. Chang W.-R.. Crystal structure of earthworm fibrinolytic enzyme component B: a novel, glycosylated two-chained trypsin. Journal of Bolecular Biology 348 (2005) 671-685.https://doi.org/10.1016/j.jmb.2005.02.055. 10.1016/j.jmb.2005.02.055 15826663

[33] Wang F. Wang C. Li M. Gui L. Zhang J. Chang W.. Crystallization and preliminary crystallographic analysis of earthworm fibrinolytic enzyme component B from Eisenia fetida. Acta Crystallographica Section D: Biological Crystallography 60 (2004) 933-935.https://doi.org/10.1107/S0907444904004895. 10.1107/S0907444904004895 15103143

[34] Tang Y. Liang D. Jiang T. Zhang J. Gui L. Chang W.. Crystal structure of earthworm fibrinolytic enzyme component a: revealing the structural determinants of its dual fibrinolytic activity. Journal of Molecular Biology 321 (2002) 57-68.https://doi.org/10.1016/S0022-2836(02)00559-4. 10.1016/S0022-2836(02)00559-4 12139933

[35] Blasi F. Vassalli J.-D. Danø K. Urokinase-type plasminogen activator: proenzyme, receptor, and inhibitors. The Journal of Cell Biology 104 (1987) 801-804.https://doi.org/10.1083/jcb.104.4.801. 10.1083/jcb.104.4.801 3031083 PMC2114431

[36] Kasai S. Arimura H. Nishida M. Suyama T.. Proteolytic cleavage of single-chain pro-urokinase induces conformational change which follows activation of the zymogen and reduction of its high affinity for fibrin. Journal of Biological Chemistry 260 (1985) 12377-12381.https://doi.org/10.1016/S0021-9258(17)39035-X. 10.1016/S0021-9258(17)39035-X 3930494

[37] Young K.-C. Shi G.-Y. Wu D.-H. Chang L.-C. Chang B.-I. Ou C.-P. Wu H.-L.. Plasminogen activation by streptokinase via a unique mechanism. Journal of Biological Chemistry 273 (1998) 3110-3116.https://doi.org/10.1074/jbc.273.5.3110. 10.1074/jbc.273.5.3110 9446629

[38] Szarka S. Sihota E. Habibi H. Wong S.-L.. Staphylokinase as a plasminogen activator component in recombinant fusion proteins. Applied and Environmental Microbiology 65 (1999) 506-513.https://doi.org/10.1128/AEM.65.2.506-513.1999. 10.1128/AEM.65.2.506-513.1999 9925575 PMC91054

[39] Ali M. Salim Hossain M. Islam M. Arman S.I. Sarwar Raju G. Dasgupta P. Noshin T.F.. Aspect of thrombolytic therapy: a review. The Scientific World Journal 2014 (2014).https://doi.org/10.1155/2014/586510. 10.1155/2014/586510 PMC4276353 25574487

[40] Verstraete M.. Third-generation thrombolytic drugs. The American Journal of Medicine 109 (2000) 52-58.https://doi.org/10.1016/S0002-9343(00)00380-6. 10.1016/S0002-9343(00)00380-6 10936478

[41] Wang W.-L. Hsu Y.-M. Lin M.-L. Chen S.-S. Lai Y.-H. Huang C.-H. Yao C.-H.. Ex Vivo Model to Evaluate the Antibacterial and Anti-Inflammatory Effects of Gelatin–Tricalcium Phosphate Composite Incorporated with Emodin and Lumbrokinase for Bone Regeneration. Bioengineering 10 (2023) 906.https://doi.org/10.3390/bioengineering10080906. 10.3390/bioengineering10080906 37627791 PMC10451264

[42] Jin L. Jin H. Zhang G. Xu G.. Changes in coagulation and tissue plasminogen activator after the treatment of cerebral infarction with lumbrokinase. Clinical Hemorheology and Microcirculation 23 (2000) 213-218. https://content.iospress.com/articles/clinical-hemorheology-and-microcirculation/ch324 11321442

[43] Zhao Y.-G. Li H. Xu W. Luo J. Xu R.-A.. An overview of the fibrinolytic enzyme from earthworm. Chinese Journal of Natural Medicines 8 (2010) 301-308. https://resource.iyp.tw/static.iyp.tw/916/files/51badbe435e29.pdf

[44] Liu J. Solanki A. White M.J. Hubbell J.A. Briquez P.S.. Therapeutic use of α2-antiplasmin as an antifibrinolytic and hemostatic agent in surgery and regenerative medicine. NPJ Regenerative Medicine 7 (2022) 34.https://doi.org/10.1038/s41536-022-00230-x. 10.1038/s41536-022-00230-x 35773290 PMC9246914

[45] Li T. Ren J. Li T. Wang Y., Advances in Applied Biotechnology: Proceedings of the 2nd International Conference on Applied Biotechnology (ICAB 2014)-Volume I, 2015, pp. 541-546.

[46] Iannucci N. Camperi S. Cascone O.. Purification of lumbrokinase from Eisenia fetida using aqueous two-phase systems and anion-exchange chromatography. Separation and Purification Technology 64 (2008) 131-134.https://doi.org/10.1016/j.seppur.2008.08.014. 10.1016/j.seppur.2008.08.014

[47] Phan T.T.B. Ta T.D. Nguyen D.T.X. Van Den Broek L.A. Duong G.T.H.. Purification and characterization of novel fibrinolytic proteases as potential antithrombotic agents from earthworm Perionyx excavatus. AMB Express 1 (2011) 26.https://doi.org/10.1186/2191-0855-1-26. 10.1186/2191-0855-1-26 21961566 PMC3210732

[48] Wu J.X. Zhao X.Y. Pan R. He R.Q.. Glycosylated trypsin-like proteases from earthworm Eisenia fetida. International Journal of Biological Macromolecules 40 (2007) 399-406.https://doi.org/10.1016/j.ijbiomac.2006.10.001. 10.1016/j.ijbiomac.2006.10.001 17113141

[49] Katheem K.S. Ibrahim M.H. Quaik S. Ahmed Ismail S. Ibrahim M.H. Quaik S. Ismail S.A.. Earthworm Based Products, Scope and Future Perspectives. Prospects of Organic Waste Management and the Significance of Earthworms (2016) 231-243.https://doi.org/10.1007/978-3-319-24708-3_10. 10.1007/978-3-319-24708-3_10

[50] Nguyen Q.T.T. Rhee H. Kim M. Lee M.Y. Lee E.-J.. Lumbrokinase, a Fibrinolytic Enzyme, Prevents Intra-Abdominal Adhesion by Inhibiting the Migrative and Adhesive Activities of Fibroblast via Attenuation of the AP-1/ICAM-1 Signaling Pathway. BioMed Research International 2023 (2023) 4050730.https://doi.org/10.1155/2023/4050730. 10.1155/2023/4050730 36685669 PMC9851794

[51] Ji H. Wang L. Bi H. Sun L. Cai B. Wang Y. Zhao J. Du Z.. Mechanisms of lumbrokinase in protection of cerebral ischemia. European Journal of Pharmacology 590 (2008) 281-289.https://doi.org/10.1016/j.ejphar.2008.05.037. 10.1016/j.ejphar.2008.05.037 18597751

[52] Hwang C. Kim D. Kim J. Huh S. *In vivo* evaluation of lumbrokinase, a fibrinolytic enzyme extracted from Lumbricus rubellus, in a prosthetic vascular graft. Journal of Cardiovascular Surgery 43 (2002) 891-894. https://www.minervamedica.it/en/journals/cardiovascular-surgery/article.php?cod=R37Y2002N06A0891 12483186

[53] Wang Y.-H. Li S.-A. Huang C.-H. Su H.-H. Chen Y.-H. Chang J.T. Huang S.-S.. Sirt1 activation by post-ischemic treatment with lumbrokinase protects against myocardial ischemia-reperfusion injury. Frontiers in Pharmacology 9 (2018) 636.https://doi.org/10.3389/fphar.2018.00636. 10.3389/fphar.2018.00636 29962953 PMC6013847

[54] Wang Y.-H. Chen K.-M. Chiu P.-S. Lai S.-C. Su H.-H. Jan M.-S. Lin C.-W. Lu D.-Y. Fu Y.-T. Liao J.-M.. Lumbrokinase attenuates myocardial ischemia-reperfusion injury by inhibiting TLR4 signaling. Journal of Molecular and Cellular Cardiology 99 (2016) 113-122.https://doi.org/10.1016/j.yjmcc.2016.08.004. 10.1016/j.yjmcc.2016.08.004 27503317

[55] Nair S.R.. Serratiopeptidase: An integrated view of multifaceted therapeutic enzyme. Biomolecules 12 (2022) 1468.https://doi.org/10.3390/biom12101468. 10.3390/biom12101468 36291677 PMC9599151

[56] Jadhav S.B. Shah N. Rathi A. Rathi V. Rathi A.. Serratiopeptidase: Insights into the therapeutic applications. Biotechnology Reports 28 (2020) e00544.https://doi.org/https://doi.org/10.1016/j.btre.2020.e00544. 10.1016/j.btre.2020.e00544 33134103 PMC7585045

[57] Salamone P.R. Wodzinski R.J.. Production, purification and characterization of a 50-kDa extracellular metalloprotease from Serratia marcescens. Applied Microbiology and Biotechnology 48 (1997) 317-324.https://doi.org/10.1007/s002530051056. 10.1007/s002530051056 9352674

[58] Miyata K. Maejima K. Tomoda K. Isono M.. Serratia protease: Part I. Purification and general properties of the enzyme. Agricultural and Biological Chemistry 34 (1970) 310-318.https://doi.org/10.1080/00021369.1970.10859598. 10.1080/00021369.1970.10859598

[59] Ethiraj S. Gopinath S.. Production, purification, characterization, immobilization, and application of Serrapeptase: a review. Frontiers in Biology 12 (2017) 333-348.https://doi.org/10.1007/s11515-017-1461-3. 10.1007/s11515-017-1461-3

[60] Raghvi A. Priya K. Balaji D.. Varied Clinical Presentations of Allergic Fungal Rhinosinusitis-A Case Series. Indian Journal of Otolaryngology and Head & Neck Surgery 75 (2023) 571-578.https://doi.org/10.1007/s12070-022-03338-0. 10.1007/s12070-022-03338-0 37275020 PMC10234982

[61] Redfern R. The ‘Miracle’Enzyme is Serrapeptase, the 2^nd^ Gift from Silkworms Giving the answer to Pain, Inflammation and Clear Arteries. Naturally Healthy Publications, 2009, ISBN 978-1910521007. https://www.amazon.com/Miracle-Enzyme-Serrapeptase-Silkworms-Inflammation/dp/1910521000/ref=monarch_sidesheet_title#

[62] Matsumoto K. Maeda H. Takata K. Kamata R. Okamura R.. Purification and characterization of four proteases from a clinical isolate of Serratia marcescens kums 3958. Journal of Bacteriology 157 (1984) 225-232.https://doi.org/10.1128/jb.157.1.225-232.1984. 10.1128/jb.157.1.225-232.1984 6418718 PMC215156

[63] Nakahama K. Yoshimura K. Marumoto R. Kikuchi M. Lee I.S. Hase T. Matsubara H.. Cloning and sequencing of Serratia protease gene. Nucleic Acids Research 14 (1986) 5843-5855.https://doi.org/10.1093/nar/14.14.5843. 10.1093/nar/14.14.5843 3016665 PMC311595

[64] Gupte V. Luthra U.. Analytical techniques for serratiopeptidase: A review. Journal of Pharmaceutical Analysis 7 (2017) 203-207.https://doi.org/10.1016/j.jpha.2017.03.005. 10.1016/j.jpha.2017.03.005 29404039 PMC5790697

[65] Tao K. Yu X. Liu Y. Shi G. Liu S. Hou T.. Cloning, expression, and purification of insecticidal protein Pr596 from locust pathogen Serratia marcescens HR-3. Current Microbiology 55 (2007) 228-233.https://doi.org/10.1007/s00284-007-0096-z. 10.1007/s00284-007-0096-z 17657528

[66] Braunagel S.C. Benedik M.J.. The metalloprotease gene of Serratia marcescens strain SM6. Molecular and General Genetics MGG 222 (1990) 446-451.https://doi.org/10.1007/BF00633854. 10.1007/BF00633854 2274043

[67] Letoffe S. Delepelaire P. Wandersman C.. Cloning and expression in Escherichia coli of the Serratia marcescens metalloprotease gene: secretion of the protease from E. coli in the presence of the Erwinia chrysanthemi protease secretion functions. Journal of Bacteriology 173 (1991) 2160-2166.https://doi.org/10.1128/jb.173.7.2160-2166.1991. 10.1128/jb.173.7.2160-2166.1991 2007544 PMC207762

[68] Moriya N. Nakata M. Nakamura M. Takaoka M. Iwasa S. Kato K. Kakinuma A.. Intestinal absorption of serrapeptase (TSP) in rats. Biotechnology and Applied Biochemistry 20 (1994) 101-108.https://doi.org/10.1111/j.1470-8744.1994.tb00308.x. 10.1111/j.1470-8744.1994.tb00308.x 7917060

[69] Bhagat S. Agarwal M. Roy V.. Serratiopeptidase: a systematic review of the existing evidence. International Journal of Surgery 11 (2013) 209-217.https://doi.org/10.1016/j.ijsu.2013.01.010. 10.1016/j.ijsu.2013.01.010 23380245

[70] Jickling G.C. Zhan X. Ander B.P. Turner R.J. Stamova B. Xu H. Tian Y. Liu D. Davis R.R. Lapchak P.A.. Genome response to tissue plasminogen activator in experimental ischemic stroke. BMC Genomics 11 (2010) 254.https://doi.org/10.1186/1471-2164-11-254. 10.1186/1471-2164-11-254 20406488 PMC2875237

[71] Mei J.f. Cai S.f. Yi Y. Wang X.d. Ying G.q.. Study of the fibrinolytic activity of serrapeptase and its in vitro thrombolytic effects. Brazilian Journal of Pharmaceutical Sciences 58 (2022) e201004.https://doi.org/10.1590/s2175-97902022e201004. 10.1590/s2175-97902022e201004

[72] Al-Khateeb T. Nusair Y.. Effect of the proteolytic enzyme serrapeptase on swelling, pain and trismus after surgical extraction of mandibular third molars. International Journal of Oral and Maxillofacial surgery 37 (2008) 264-268.https://doi.org/10.1016/j.ijom.2007.11.011. 10.1016/j.ijom.2007.11.011 18272344

[73] Jadav S.P. Patel N.H. Shah T.G. Gajera M.V. Trivedi H.R. Shah B.K.. Comparison of antiinflammatory activity of serratiopeptidase and diclofenac in albino rats. Journal of Pharmacology and Pharmacotherapeutics 1 (2010) 116-117.https://doi.org/10.4103/0976-500X.72362. 10.4103/0976-500X.72362 21350623 PMC3043339

[74] Suma K. Manasa H. Likhitha A. Nagamani T.. Isolation, Purification, and Characterization of Serratiopeptidase Enzyme from Serratia marcescens. Int. J. Innov. Sci. Res. Technol 5 (2020) 156-161.https://doi.org/10.38124/IJISRT20JUL135. 10.38124/IJISRT20JUL135

[75] Kumar D. Verma D. Abbot V.. A review on pharmaceutical, pharmacological and chemical aspects of serratiopeptidase as anti-inflammatory agent. Materials Today: Proceedings (2023).https://doi.org/10.1016/j.matpr.2023.01.256. 10.1016/j.matpr.2023.01.256

[76] Krishna B.P. Reddy B.P. Kumar D.Y. Ummar M. Shekhar V. Tiwari R.V.C.. Role of serratiopeptidase and dexamethasone in the control of postoperative swelling. Annals of Maxillofacial Surgery 10 (2020) 108-113.https://doi.org/10.4103/ams.ams_249_19. 10.4103/ams.ams_249_19 32855925 PMC7433958

[77] Yadav V. Sharma S. Kumar A. Singh S. Ravichandiran V.. Serratiopeptidase Attenuates Lipopolysaccharide-Induced Vascular Inflammation by Inhibiting the Expression of Monocyte Chemoattractant Protein-1. Current Issues in Molecular Biology 45 (2023) 2201-2212.https://doi.org/10.3390/cimb45030142. 10.3390/cimb45030142 36975512 PMC10047379

[78] Menon V.D. Muthusekhar M.. Effectiveness of anti-inflammatory properties of combination of bromelain, trypsin and rutoside with combination of diclofenac and serratiopeptidase following surgical removal of impacted mandibular third molar-a randomised double blinded clinical trial. Int J Dentistry Oral Sci 8 (2021) 4217-4221.https://doi.org/dx.doi.org/10.19070/2377-8075-21000859. 10.19070/2377-8075-21000859

[79] Mammdoh J.K. Al-Alsadoon L.H. Taqa G.A. Taqa A.A.. Evaluation of Anti-inflammatory Effect of Topical Serratiopeptidase in Mice. Inflammation 12 (2022) 13.https://doi.org/10.25258/ijddt.12.1.29. 10.25258/ijddt.12.1.29

[80] Mazzone A. Catalani M. Costanzo M. Drusian A. Mandoli A. Russo S. Guarini E. Vesperini G.. Evaluation of Serratia peptidase in acute or chronic inflammation of otorhinolaryngology pathology: a multicentre, double-blind, randomized trial versus placebo. Journal of International Medical Research 18 (1990) 379-388.https://doi.org/10.1177/0300060590018005. 10.1177/0300060590018005 2257960

[81] Dhiman A. Purohit R.. Identification of potential mutational hotspots in serratiopeptidase to address its poor pH tolerance issue. Journal of Biomolecular Structure and Dynamics 41 (2023) 8831-8843.https://doi.org/10.1080/07391102.2022.2137699. 10.1080/07391102.2022.2137699 36307910

[82] Dhiman A. Purohit R.. Targeting tachykinin peptides involved in viral infections through in silico approach: Screening the unforeseen potency of serratiopeptidase. Journal of Molecular Liquids 392 (2023) 123504.https://doi.org/10.1016/j.molliq.2023.123504. 10.1016/j.molliq.2023.123504

[83] Gupta K.K. Rahman A. Kumar A. Gavel P. Asia P.. Adjuvant therapy with Serratiopeptidase and Vitamin D for COVID-19 patients: A new perspective. Int. J. Med. Sci 4 (2021) 282-287. https://www.researchgate.net/publication/352282548_Adjuvant_therapy_with_Serratiopeptidase_and_Vitamin_D_for_COVID-19_patients_A_new_perspective

[84] Kase Y. Seo H. Oyama Y. Sakata M. Tomoda K. Takahama K. Hitoshi T. Okano Y. Miyata T.. A new method for evaluating mucolytic expectorant activity and its application. II. Application to two proteolytic enzymes, serratiopeptidase and seaprose. Arzneimittel-forschung 32 (1982) 374-378. https://europepmc.org/article/med/7049188 7049188

[85] Gioia M. Ciaccio C. Calligari P. De Simone G. Sbardella D. Tundo G. Fasciglione G.F. Di Masi A. Di Pierro D. Bocedi A.. Role of proteolytic enzymes in the COVID-19 infection and promising therapeutic approaches. Biochemical Pharmacology 182 (2020) 114225.https://doi.org/10.1016/j.bcp.2020.114225. 10.1016/j.bcp.2020.114225 32956643 PMC7501082

[86] Gomathy V. Manigandan V. Vignesh N. Thabitha A. Saravanan R.. Evaluation of antibacterial, teratogenicity and antibiofilm effect of sulfated chitosans extracted from marine waste against microorganism. Journal of Bioactive and Compatible Polymers 36 (2021) 249-258.https://doi.org/10.1177/08839115211014225. 10.1177/08839115211014225

[87] Selan L. Papa R. Tilotta M. Vrenna G. Carpentieri A. Amoresano A. Pucci P. Artini M.. Serratiopeptidase: a well-known metalloprotease with a new non-proteolytic activity against S. aureus biofilm. BMC Microbiology 15 (2015) 207 .https://doi.org/10.1186/s12866-015-0548-8. 10.1186/s12866-015-0548-8 26453184 PMC4600273

[88] Devi C.S. Elizabeth Joseph R. Saravanan H. Naine S.J. Srinivansan V.M.. Screening and molecular characterization of Serratia marcescens VITSD2: A strain producing optimum serratiopeptidase. Frontiers in Biology 8 (2013) 632-639.https://doi.org/10.1007/s11515-013-1284-9. 10.1007/s11515-013-1284-9

[89] Mecikoglu M. Saygi B. Yildirim Y. Karadag-Saygi E. Ramadan S.S. Esemenli T.. The effect of proteolytic enzyme serratiopeptidase in the treatment of experimental implant-related infection. JBJS 88 (2006) 1208-1214.https://doi.org/10.2106/JBJS.E.00007. 10.2106/JBJS.E.00007 16757752

[90] Vidmar B. Vodovnik M.. Microbial keratinases: enzymes with promising biotechnological applications. Food Technology and Biotechnology 56 (2018) 312-328.https://doi.org/10.17113/ftb.56.03.18.5658. 10.17113/ftb.56.03.18.5658 30510475 PMC6233012

[91] Nnolim N.E. Udenigwe C.C. Okoh A.I. Nwodo U.U.. Microbial keratinase: Next generation green catalyst and prospective applications. Frontiers in Microbiology 11 (2020) 580164.https://doi.org/10.3389/fmicb.2020.580164. 10.3389/fmicb.2020.580164 33391200 PMC7775373

[92] Prusiner S.B.. Prions. Proceedings of the National Academy of Sciences 95 (1998) 13363-13383.https://doi.org/10.1073/pnas.95.23.13363. 10.1073/pnas.95.23.13363 PMC33918 9811807

[93] Prusiner S.B.. Novel proteinaceous infectious particles cause scrapie. Science 216 (1982) 136-144.https://doi.org/10.1126/science.6801762. 10.1126/science.6801762 6801762

[94] Ascari L.M. Rocha S.C. Gonçalves P.B. Vieira T.C. Cordeiro Y.. Challenges and advances in antemortem diagnosis of human transmissible spongiform encephalopathies. Frontiers in Bioengineering and Biotechnology 8 (2020) 585896.https://doi.org/10.3389/fbioe.2020.585896. 10.3389/fbioe.2020.585896 33195151 PMC7606880

[95] Rutala W.A. Weber D.J.. Guideline for disinfection and sterilization of prion-contaminated medical instruments. Infection Control & Hospital Epidemiology 31 (2010) 107-117.https://doi.org/10.1086/650197. 10.1086/650197 20055640

[96] Taylor D.. Inactivation of transmissible degenerative encephalopathy agents: a review. The Veterinary Journal 159 (2000) 10-17.https://doi.org/10.1053/tvjl.1999.0406. 10.1053/tvjl.1999.0406 10640408

[97] Gupta R. Sharma R. Beg Q.K.. Revisiting microbial keratinases: next generation proteases for sustainable biotechnology. Critical Reviews in Biotechnology 33 (2013) 216-228.https://doi.org/10.3109/07388551.2012.685051. 10.3109/07388551.2012.685051 22642703

[98] Kamaraj S. Vuppu S.. In-silico study of bacterial keratinase and optimization of extraction procedure for keratin from Country chicken and Indian blue rock pigeon feathers. Kuwait Journal of Science 51 (2024) 100149.https://doi.org/10.1016/j.kjs.2023.10.016. 10.1016/j.kjs.2023.10.016

[99] Ningthoujam D.S. Mukherjee S. Devi L.J. Singh E.S. Tamreihao K. Khunjamayum R. Banerjee S. Mukhopadhyay D.. In vitro degradation of β-amyloid fibrils by microbial keratinase. Alzheimer's & Dementia: Translational Research & Clinical Interventions 5 (2019) 154-163.https://doi.org/10.1016/j.trci.2019.03.003. 10.1016/j.trci.2019.03.003 PMC6527806 31193333

[100] Yoshioka M. Miwa T. Horii H. Takata M. Yokoyama T. Nishizawa K. Watanabe M. Shinagawa M. Murayama Y.. Characterization of a proteolytic enzyme derived from a Bacillus strain that effectively degrades prion protein. Journal of Applied Microbiology 102 (2007) 509-515.https://doi.org/10.1111/j.1365-2672.2006.03080.x. 10.1111/j.1365-2672.2006.03080.x 17241357

[101] Okoroma E.A. Purchase D. Garelick H. Morris R. Neale M.H. Windl O. Abiola O.O.. Enzymatic formulation capable of degrading scrapie prion under mild digestion conditions. PloS One 8 (2013) e68099.https://doi.org/10.1371/journal.pone.0068099. 10.1371/journal.pone.0068099 23874511 PMC3712960

[102] Langeveld J.P. Wang J.-J. Van de Wiel D.F. Shih G.C. Garssen G.J. Bossers A. Shih J.C.. Enzymatic degradation of prion protein in brain stem from infected cattle and sheep. The Journal of Iinfectious Diseases 188 (2003) 1782-1789.https://doi.org/10.1086/379664. 10.1086/379664 14639552

[103] Purchase D.. Microbial keratinases: Characteristics, biotechnological applications and potential. The handbook of microbial bioresources. Wallingford: CAB International Publishing (2016) 634-674.https://doi.org/10.1079/9781780645216.0634. 10.1079/9781780645216.0634

[104] Hui Z. Doi H. Kanouchi H. Matsuura Y. Mohri S. Nonomura Y. Oka T.. Alkaline serine protease produced by Streptomyces sp. degrades PrPSc. Biochemical and Biophysical Research Communications 321 (2004) 45-50.https://doi.org/10.1016/j.bbrc.2004.06.100. 10.1016/j.bbrc.2004.06.100 15358213

[105] Sharma R. Gupta R.. Coupled action of γ-glutamyl transpeptidase-glutathione and keratinase effectively degrades feather keratin and surrogate prion protein, Sup 35NM. Bioresource Technology 120 (2012) 314-317. https://doi.org/10.1016/j.biortech.2012.06.038 10.1016/j.biortech.2012.06.038 22776236

[106] Nishinari K. Fang Y. Nagano T. Guo S. Wang R., *Soy as a food ingredient*, in *Proteins in food processing*, Yada R.Y. (Ed.), Elsevier, 2018, p. 149-186.https://doi.org/10.1016/B978-0-08-100722-8.00007-3. 10.1016/B978-0-08-100722-8.00007-3

[107] Gallelli G. Di Mizio G. Palleria C. Siniscalchi A. Rubino P. Muraca L. Cione E. Salerno M. De Sarro G. Gallelli L.. Data recorded in real life support the safety of nattokinase in patients with vascular diseases. Nutrients 13 (2021) 2031.https://doi.org/10.3390/nu13062031. 10.3390/nu13062031 34199189 PMC8231931

[108] Yokoyama T. Nakamura T. Kimijima M. Mandokoro K. Tokumaru M. Takatsuka A. Narisawa N. Kobayashi R. Takenaga F.. Subtilisin NAT, a subtilisin-like serine protease present in fermented soybean “natto” extract, inhibits Streptococcus mutans biofilm formation. Food Science and Technology Research 27 (2021) 537-542.https://doi.org/10.3136/fstr.27.537. 10.3136/fstr.27.537

[109] Kamata H. Yamagata Y. Nakamura T. Nakajima T. Oda K. Murao S. Ichishima E.. Characterization of the complex between α2-macroglobulin and a serine proteinase from Bacillus natto. Agricultural and Biological Chemistry 53 (1989) 2695-2702.https://doi.org/10.1080/00021369.1989.10869721. 10.1080/00021369.1989.10869721

[110] Fujita M. Nomura K. Hong K. Ito Y. Asada A. Nishimuro S.. Purification and characterization of a strong fibrinolytic enzyme (nattokinase) in the vegetable cheese natto, a popular soybean fermented food in Japan. Biochemical and Bbiophysical Research Communications 197 (1993) 1340-1347.https://doi.org/10.1006/bbrc.1993.2624. 10.1006/bbrc.1993.2624 8280151

[111] Cai D. Zhu C. Chen S.. Microbial production of nattokinase: current progress, challenge and prospect. World Journal of Microbiology and Biotechnology 33 (2017) 84.https://doi.org/10.1007/s11274-017-2253-2. 10.1007/s11274-017-2253-2 28378222

[112] Selvarajan E. Bhatnagar N.. Nattokinase: an updated critical review on challenges and perspectives. Cardiovascular & Hematological Agents in Medicinal Chemistry (Formerly Current Medicinal Chemistry-Cardiovascular & Hematological Agents) 15 (2017) 128-135.https://doi.org/10.2174/1871525716666171207153332. 10.2174/1871525716666171207153332 29219060

[113] Fujita M. Hong K. Ito Y. Fujii R. Kariya K. Nishimuro S.. Thrombolytic effect of nattokinase on a chemically induced thrombosis model in rat. Biological and Pharmaceutical Bulletin 18 (1995) 1387-1391.https://doi.org/10.1248/bpb.18.1387. 10.1248/bpb.18.1387 8593442

[114] Wang L. Meng J. Yu X. Wang J. Zhang Y. Zhang M. Zhang Y. Wang H. Feng H. Tian Q.. Construction of highly active and stable recombinant nattokinase by engineered bacteria and computational design. Archives of Biochemistry and Biophysics 760 (2024) 110126.https://doi.org/10.1016/j.abb.2024.110126. 10.1016/j.abb.2024.110126 39154817

[115] Chen C. Ai Q.-D. Chu S.-F. Zhang Z. Chen N.-H.. NK cells in cerebral ischemia. Biomedicine & Pharmacotherapy 109 (2019) 547-554.https://doi.org/10.1016/j.biopha.2018.10.103. 10.1016/j.biopha.2018.10.103 30399590

[116] Wang J.-M. Chen H.-Y. Cheng S.-M. Chen S.-H. Yang L.-L. Cheng F.-C.. Nattokinase reduces brain infarction, fibrinogen and activated partial thromboplastin time against cerebral ischemia-reperfusion injury. Journal of Food and Drug Analysis 20 (2012) 1. https://doi.org/10.6227/jfda.2012200317 10.6227/jfda.2012200317

[117] Hsia C.-H. Shen M.-C. Lin J.-S. Wen Y.-K. Hwang K.-L. Cham T.-M. Yang N.-C.. Nattokinase decreases plasma levels of fibrinogen, factor VII, and factor VIII in human subjects. Nutrition Research 29 (2009) 190-196.https://doi.org/10.1016/j.nutres.2009.01.009. 10.1016/j.nutres.2009.01.009 19358933

[118] Guangbo Y. Min S. Wei S. Lixin M. Chao Z. Yaping W. Zunxi H.. Heterologous expression of nattokinase from B. subtilis natto using Pichia pastoris GS115 and assessment of its thrombolytic activity. BMC Biotechnology 21 (2021) 49.https://doi.org/10.1186/s12896-021-00708-4. 10.1186/s12896-021-00708-4 34372833 PMC8353737

[119] Krishnamurthy A. Belur P.D. Subramanya S.B.. Methods available to assess therapeutic potential of fibrinolytic enzymes of microbial origin: a review. Journal of Analytical Science and Technology 9 (2018) 10.https://doi.org/10.1186/s40543-018-0143-3. 10.1186/s40543-018-0143-3

[120] Pinontoan R. Sanjaya A. Jo J.. Fibrinolytic characteristics of Bacillus subtilis G8 isolated from natto. Bioscience of Microbiota, Food and Health 40 (2021) 144-149.https://doi.org/10.12938/bmfh.2020-071. 10.12938/bmfh.2020-071 34285859 PMC8279889

[121] Kamiya S. Hagimori M. Ogasawara M. Arakawa M.. In vivo evaluation method of the effect of nattokinase on carrageenan-induced tail thrombosis in a rat model. Acta Haematologica 124 (2010) 218-224.https://doi.org/10.1007/s10930-021-10023-8. 10.1007/s10930-021-10023-8 21071931

[122] Taniguchi M. Aida R. Saito K. Kikura T. Ochiai A. Saitoh E. Tanaka T.. Identification and characterization of multifunctional cationic peptides from enzymatic hydrolysates of soybean proteins. Journal of Bioscience and Bioengineering 129 (2020) 59-66.https://doi.org/10.1016/j.jbiosc.2019.06.016. 10.1016/j.jbiosc.2019.06.016 31324383

[123] Liu S. Zhu J. Liu C. Li J. Li Z. Zhao J. Liu H.. Synthesis of sustained release/controlled release nanoparticles carrying nattokinase and their application in thrombolysis. Die Pharmazie-An International Journal of Pharmaceutical Sciences 76 (2021) 145-149.https://doi.org/10.1691/ph.2021.0155. 10.1691/ph.2021.0155 33849698

[124] Yatagai C. Maruyama M. Kawahara T. Sumi H.. Nattokinase-promoted tissue plasminogen activator release from human cells. Pathophysiology of Haemostasis and Thrombosis 36 (2007) 227-232.https://doi.org/10.1159/000252817. 10.1159/000252817 19996631

[125] Wu H. Wang Y. Zhang Y. Xu F. Chen J. Duan L. Zhang T. Wang J. Zhang F.. Breaking the vicious loop between inflammation, oxidative stress and coagulation, a novel anti-thrombus insight of nattokinase by inhibiting LPS-induced inflammation and oxidative stress. Redox Biology 32 (2020) 101500.https://doi.org/10.1016/j.redox.2020.101500. 10.1016/j.redox.2020.101500 32193146 PMC7078552

[126] Zhou L. Hao N. Li X. Chen J. Yang R. Song C. Sun Y. Zhang Q.. Nattokinase mitigated dextran sulfate sodium-induced chronic colitis by regulating microbiota and suppressing tryptophan metabolism via inhibiting IDO-1. Journal of Functional Foods 75 (2020) 104251.https://doi.org/10.1016/j.jff.2020.104251. 10.1016/j.jff.2020.104251

[127] Huang Z. Ng T.K. Chen W. Sun X. Huang D. Zheng D. Yi J. Xu Y. Zhuang X. Chen S.. Nattokinase attenuates retinal neovascularization via modulation of Nrf2/HO-1 and glial activation. Investigative Ophthalmology & Visual Science 62 (2021) 25-25.https://doi.org/10.1167/iovs.62.6.25. 10.1167/iovs.62.6.25 PMC8164371 34036312

[128] Elbakry M.M. Mansour S.Z. Helal H. Ahmed E.S.. Nattokinase attenuates bisphenol A or gamma irradiation-mediated hepatic and neural toxicity by activation of Nrf2 and suppression of inflammatory mediators in rats. Environmental Science and Pollution Research 29 (2022) 75086-75100.https://doi.org/10.1007/s11356-022-21126-9. 10.1007/s11356-022-21126-9 35648353 PMC9550699

[129] Chou H.-Y. Liu L.-H. Chen C.-Y. Lin I.-F. Ali D. Lee A.Y.-L. Wang H.-M.D. Bifunctional mechanisms of autophagy and apoptosis regulations in melanoma from Bacillus subtilis natto fermentation extract. Food and Chemical Toxicology 150 (2021) 112020.https://doi.org/10.1016/j.fct.2021.112020. 10.1016/j.fct.2021.112020 33513408

[130] Song Y. Yu J. Song J. Wang S. Cao T. Liu Z. Gao X. Wei Y.. The antihypertensive effect and mechanisms of bioactive peptides from Ruditapes philippinarum fermented with Bacillus natto in spontaneously hypertensive rats. Journal of Functional Foods 79 (2021) 104411.https://doi.org/10.1016/j.jff.2021.104411. 10.1016/j.jff.2021.104411

[131] Keziah S.M. Devi C.S.. Fibrinolytic and ACE Inhibitory Activity of Nattokinase Extracted from Bacillus subtilis VITMS 2: A Strain Isolated from Fermented Milk of Vigna unguiculata. The Protein Journal 40 (2021) 876-890.https://doi.org/10.1007/s10930-021-10023-8. 10.1007/s10930-021-10023-8 34611797

[132] Metkar S.K. Girigoswami A. Murugesan R. Girigoswami K.. In vitro and in vivo insulin amyloid degradation mediated by Serratiopeptidase. Materials Science and Engineering: C 70 (2017) 728-735.https://doi.org/10.1016/j.msec.2016.09.049. 10.1016/j.msec.2016.09.049 27770948

[133] Metkar S.K. Ghosh S. Girigoswami A. Girigoswami K.. Prion peptide 106-126 degradation potential of Serratiopetidase and Lumbrokinase-an in vitro and in silico perspective. CNS & Neurological Disorders-Drug Targets (Formerly Current Drug Targets-CNS & Neurological Disorders) 18 (2019) 723-731.https://doi.org/10.2174/1871527318666191021150002. 10.2174/1871527318666191021150002 31642793

[134] Metkar S.K. Girigoswami A. Bondage D.D. Shinde U.G. Girigoswami K.. The potential of lumbrokinase and serratiopeptidase for the degradation of Aβ 1–42 peptide–an in vitro and in silico approach. International Journal of Neuroscience 134 (2022) 112-123.https://doi.org/10.1080/00207454.2022.2089137. 10.1080/00207454.2022.2089137 35694981

[135] Metkar S.K. Girigoswami A. Murugesan R. Girigoswami K.. Lumbrokinase for degradation and reduction of amyloid fibrils associated with amyloidosis. Journal of Applied Biomedicine 15 (2017) 96-104.https://doi.org/10.1016/j.jab.2017.01.003. 10.1016/j.jab.2017.01.003

[136] Metkar S.K. Girigoswami A. Vijayashree R. Girigoswami K.. Attenuation of subcutaneous insulin induced amyloid mass in vivo using Lumbrokinase and Serratiopeptidase. International Journal of Biological Macromolecules 163 (2020) 128-134.https://doi.org/10.1016/j.ijbiomac.2020.06.256. 10.1016/j.ijbiomac.2020.06.256 32615214

[137] Velander P. Wu L. Henderson F. Zhang S. Bevan D.R. Xu B.. Natural product-based amyloid inhibitors. Biochemical Pharmacology 139 (2017) 40-55.https://doi.org/10.1016/j.bcp.2017.04.004. 10.1016/j.bcp.2017.04.004 28390938 PMC5841551

[138] Ciccone L. Tonali N. Nencetti S. Orlandini E.. Natural compounds as inhibitors of transthyretin amyloidosis and neuroprotective agents: Analysis of structural data for future drug design. Journal of Enzyme Inhibition and Medicinal Chemistry 35 (2020) 1145-1162.https://doi.org/10.1080/14756366.2020.1760262. 10.1080/14756366.2020.1760262 32419519 PMC7301710

[139] Cummings J. Lee G. Ritter A. Zhong K.. Alzheimer's disease drug development pipeline: 2018. Alzheimer's & Dementia: Translational Research & Clinical Interventions 4 (2018) 195-214.https://doi.org/10.1016/j.trci.2018.03.009. 10.1016/j.trci.2018.03.009 29955663 PMC6021548

[140] Sevigny J. Chiao P. Bussière T. Weinreb P.H. Williams L. Maier M. Dunstan R. Salloway S. Chen T. Ling Y.. The antibody aducanumab reduces Aβ plaques in Alzheimer’s disease. Nature 537 (2016) 50-56.https://doi.org/10.1038/nature19323. 10.1038/nature19323 27582220

[141] Guthrie H. Honig L.S. Lin H. Sink K.M. Blondeau K. Quartino A. Dolton M. Carrasco-Triguero M. Lian Q. Bittner T.. Safety, tolerability, and pharmacokinetics of crenezumab in patients with mild-to-moderate Alzheimer’s disease treated with escalating doses for up to 133 weeks. Journal of Alzheimer's disease 76 (2020) 967-979.https://doi.org/10.3233/JAD-200134. 10.3233/JAD-200134 PMC7505005 32568196

[142] Cantillon M. Andreasen N. Prins N.. Phase 1/2a intravenous and subcutaneous oligomer-specific antibody KHK6640 in mild to moderate Alzheimer’s disease. The Journal of Prevention of Alzheimer's Disease 11 (2024) 65-70.https://doi.org/10.14283/jpad.2024.2. 10.14283/jpad.2024.2 38230718

[143] Davtyan H. Ghochikyan A. Petrushina I. Hovakimyan A. Davtyan A. Poghosyan A. Marleau A.M. Movsesyan N. Kiyatkin A. Rasool S.. Immunogenicity, efficacy, safety, and mechanism of action of epitope vaccine (Lu AF20513) for Alzheimer's disease: prelude to a clinical trial. Journal of Neuroscience 33 (2013) 4923-4934.https://doi.org/10.1523/JNEUROSCI.4672-12.2013. 10.1523/JNEUROSCI.4672-12.2013 23486963 PMC3634356

[144] Lowe S.L. Willis B.A. Hawdon A. Natanegara F. Chua L. Foster J. Shcherbinin S. Ardayfio P. Sims J.R.. Donanemab (LY3002813) dose-escalation study in Alzheimer's disease. Alzheimer's & Dementia: Translational Research & Clinical Interventions 7 (2021) e12112.https://doi.org/10.1002/trc2.12112. 10.1002/trc2.12112 33614890 PMC7882532

[145] Swanson C.J. Zhang Y. Dhadda S. Wang J. Kaplow J. Lai R.Y. Lannfelt L. Bradley H. Rabe M. Koyama A.. A randomized, double-blind, phase 2b proof-of-concept clinical trial in early Alzheimer’s disease with lecanemab, an anti-Aβ protofibril antibody. Alzheimer's Research & Therapy 13 (2021) 80.https://doi.org/10.1186/s13195-021-00813-8. 10.1186/s13195-021-00813-8 PMC8053280 33865446

[146] Matijevic M. Watanabe H. Sato Y. Bernier F. McGrath S. Burns L. Yamamoto N. Ogo M. Dezso Z. Chow J.. P4–163: a single dose of the beta-secretase inhibitor, e2609, decreases CSF bace1 enzymatic activity in cynomolgus monkeys. Alzheimer's & Dementia 11 (2015) P841.https://doi.org/10.1016/j.jalz.2015.06.1870. 10.1016/j.jalz.2015.06.1870

[147] Dodel R. Rominger A. Blennow K. Barkhof F. Wietek S. Haag S. Bartenstein P. Farlow M. Jessen F.. P4-411: A randomized, double-blind, placebo-controlled dose-finding trial of intravenous immunoglobulin (IVIG; Octagam® 10%, Octapharma AG) in patients with mild to moderate Alzheimer's disease (GAM10-04). Alzheimer's & Dementia 7 (2011) e55-e56.https://doi.org/10.1016/j.jalz.2011.09.107. 10.1016/j.jalz.2011.09.107

[148] Yu H.J. Dickson S.P. Wang P.-N. Chiu M.-J. Huang C.-C. Chang C.-C. Liu H. Hendrix S.B. Dodart J.-C. Verma A. Safety, tolerability, immunogenicity, and efficacy of UB-311 in participants with mild Alzheimer's disease: a randomised, double-blind, placebo-controlled, phase 2a study. EBioMedicine 94 (2023) 104665.https://doi.org/10.1016/j.ebiom.2023.104665. 10.1016/j.ebiom.2023.104665 37392597 PMC10338203

[149] Neumann U. Ufer M. Jacobson L.H. Rouzade-Dominguez M.L. Huledal G. Kolly C. Lüönd R.M. Machauer R. Veenstra S.J. Hurth K.. The BACE-1 inhibitor CNP 520 for prevention trials in Alzheimer's disease. EMBO Molecular Medicine 10 (2018) e9316.https://doi.org/10.15252/emmm.201809316. 10.15252/emmm.201809316 30224383 PMC6220303

[150] Jacobsen H. Ozmen L. Caruso A. Narquizian R. Hilpert H. Jacobsen B. Terwel D. Tanghe A. Bohrmann B.. Combined treatment with a BACE inhibitor and anti-Aβ antibody gantenerumab enhances amyloid reduction in APPLondon mice. Journal of Neuroscience 34 (2014) 11621-11630.https://doi.org/10.1523/JNEUROSCI.1405-14.2014. 10.1523/JNEUROSCI.1405-14.2014 25164658 PMC4145168

[151] Moussa C.E.. Beta-secretase inhibitors in phase I and phase II clinical trials for Alzheimer’s disease. Expert Opinion on Investigational Drugs 26 (2017) 1131-1136.https://doi.org/10.1080/13543784.2017.1369527. 10.1080/13543784.2017.1369527 28817311

[152] Abushouk A.I. Elmaraezy A. Aglan A. Salama R. Fouda S. Fouda R. AlSafadi A.M.. Bapineuzumab for mild to moderate Alzheimer’s disease: a meta-analysis of randomized controlled trials. BMC Neurology 17 (2017) 66.https://doi.org/10.1186/s12883-017-0850-1. 10.1186/s12883-017-0850-1 28376794 PMC5381133

